# Opening the black box of transfer systems in public sector health services in a Western state in India

**DOI:** 10.1186/s12913-016-1675-0

**Published:** 2016-08-22

**Authors:** Bhaskar Purohit, Tim Martineau, Kabir Sheikh

**Affiliations:** 1 Indian Institute of Public Health Gandhinagar (IIPHG), Sardar Patel Institute Campus, Drive in Road, Thaltej Ahmedabad, 380054 India; 2 Liverpool School of Tropical Medicine (LSTM), Pembroke Place, Liverpool, L3 5QA UK; 3 Public Health Foundation of India, Plot No. 47, Sector 44, Institutional Area Gurgaon, 122002 India

## Abstract

**Background:**

Limited research on Posting and Transfer (P&T) policies and systems in the public sector health services and the reluctance for an open debate on the issue makes P&T as a black box. Limited research on P&T in India suggests that P&T policies and systems are either non-existent, weak, poorly implemented or characterized by corruption. Hence the current study aimed at opening the “black box” of P&T systems in public sector health services in India by assessing the implementation gaps between P&T policies and their actual implementation.

**Methods:**

This was a qualitative study carried out in Department of Health, in a Western State in India. To understand the extant P&T policies, a systems map was first developed with the help of document review and Key Informant (KI) Interviews. Next systems audit was carried out to assess the actual implementation of transfer policies by interviewing Medical Officers (MOs), the group mainly affected by the P&T policies. Job histories were constructed from the interviews to understand transfer processes like frequencies of transfers and to assess if transfer rules were adhered. The analysis is based on a synthesis of document review, 19 in-depth interviews with MOs working with state health department and five in-depth interviews with Key Informants (KIs). Framework analysis approach was used to analyze data using NVIVO.

**Results:**

The state has a generic transfer guideline applicable to all government officers but there is no specific transfer policy or guideline for government health personnel. The generic transfer guidelines are weakly implemented indicating a significant gap between policy and actual implementation. The formal transfer guidelines are undermined by a parallel system in which desirable posts are attained, retained or sometimes given up by the use of political connections and money. MOs’ experiences of transfers were marked by perceptions of unfairness and irregularities reflected through interviews as well as the job histories.

**Discussion:**

The generic transfer rules and ambiguity in how transfers are treated may explain the discrepancy between policy and implementation leading to systems abuse. This discrepancy could have negative influence on MOs’ morale which could in turn affect distribution of MOs. Where possible, ambiguity in the rules should be avoided and a greater transparency on implementation of the transfer rules is needed. However, it may not be possible to make any significant improvements to P&T policies and how they are implemented until the external pressure that creates parallel systems is greatly reduced in translating HR policy into HR practice.

**Conclusions:**

Effective P&T policies and implementation may have important implications for organizational performance and may help to improve Human Resource (HR) policy and HR expertise. Also there is a greater need for transparency on implementation of the rules. However, it may not be possible to make any significant improvements to P&T policies and how they are implemented until the external pressure that creates parallel systems is greatly reduced.

## Background

Shortage and inequitable distribution of doctors serving in rural and underserved areas remains a major problem in many countries including India [1] with higher concentration in urban areas [2, 3]. The issue of shortage is linked to poor coverage of basic health services in several countries [1] and poor quality of care being provided to people [4]. Poor health outcomes are often caused by supply side delivery problems such as absenteeism and overall low productivity of Human Resources for Health [1, 5].

Posting and Transfer (P&T) is a mechanism to ensure adequate and equitable staffing across the services and locations. But there is a dearth of literature and reluctance for open debate on P&T in public sector health services in India and elsewhere. Hence the issue of P&T is such where although parallel systems emerge openly within the systems yet there is not enough empirical evidence on the issue [6]. Poor P&T practices have been documented as a cause of low morale, geographical misdistribution and migration of health workers [7]. Further, poor P&T policies directly or indirectly prevent or discourage healthcare providers from either joining Public Health Sector or such policies contribute to provider’s dissatisfaction with the existing system and may lead to low morale, poor performance, high absenteeism and attrition [8, 9]. Similarly, lack of adherence to the policies or bypassing them and creating non-transparent and alternative systems of Human Resource Management (HRM) systems can lead to corruption and reduce worker morale thereby affecting the overall effectiveness of the system [10]. However, the effect of the P&T policies on workforce availability is not only dependent on existence of policies but also dependent on the way in which P&T policies are implemented. Hence effectiveness of such policies depends on the degree of “implementation fidelity” of the relevant HR systems [11].

Boxall and Macky [12] refer to the importance of not only having the Human Resource Management (HRM) policies in place but also the way HRM policies are implemented. The Boxall and Macky [12] framework suggests a causal link of between intended Human Resource (HR) practices and the way they are implemented by managers; the perceptions and behaviour of employees; and organisational performance.

Literature on P&T suggests that systems with weak ‘implementation fidelity’ or non-compliance with P&T rules may lead to systems abuse and corruption [8, 13, 14]. La Forgia el al [15] suggest that the mis-use of formal systems come from the use of parallel systems driven externally e,g through political interference, and internally by health worker preferences. The lack of organizational governance means that the implementation of policies can be influenced by externally and internally driven pressures [16]. The current study focuses on de facto institutional and governance arrangements relating to P&T.

**Fig. 1:**
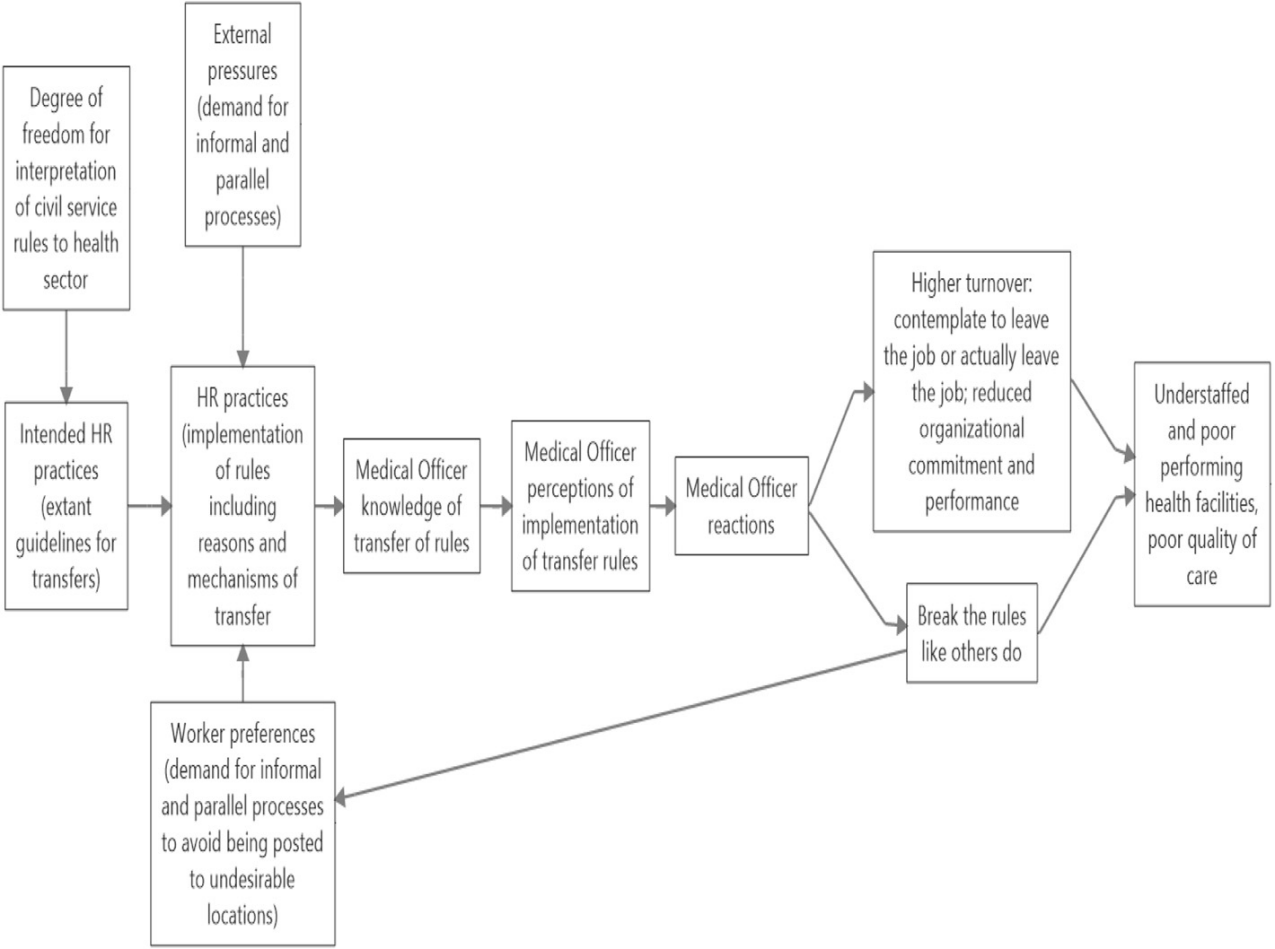
Conceptual framework for the study. Source: Based on Boxall and Macky [12] and La Forgia et al. [15]

Based on the work of Boxall and Macky [12] and La Forgia et al. [15] we developed a framework that draws a link between intended HR practices, actual HR practices, HR practices as perceived by Medical Officers (MOs), MOs reactions and its potential link to staff turnover (behavior) and organisational performance. The conceptual framework, shown in Fig. 1, also draws from Forgia et al. to explain how external pressures and worker preferences lead to systems abuse.

Anecdotal evidence and limited literature suggest that HRM policies on P&T in the government health sector in India are non-existent, lack transparency or are poorly implemented. The poor implementation of transfer policies results in lack of staff at health facilities, staff not living at posted centres, high absenteeism and political interference [17]. The evidence from other countries indicates that HRM systems in public sector can be inefficient and slow [16, 18]. India also experiences a big variation in the way in which HRM systems are governed and policies are implemented across the country. Hence to address the issue of rational distribution of health personnel, there is a greater need to examine HR policies and practices relating to transfer of Medical Officers (MOs) or doctors in the state.

The P&T system in the government health sector in Pakistan has also been described as arbitrary and while such systems may offer security and stability in the job, the frequent and often inconsistent transfers make such jobs unstable at the same time [8]. Schaaf et al. [6] describe transfers in the health sector as being ‘Mission Inconsistent’, as they neither maximize health outcomes nor respect the norms of health care worker professionalism [6]. Several studies have found that corruption in the process of transfers is a pervasive problem affecting not only the health sector but it also affects individuals [19–22].

With this backdrop the main objectives of the study were to document extant transfer policies and assess how transfer policies are implemented in practice to understand discrepancies between policies and their actual implementation. The main aim of the paper is to shine a light into the black box of P&T systems in public sector health services in a Western state in India. Although there have been a few studies conducted in other countries that aimed at understanding the issue of P&T from the view point of corruption, to our knowledge this is one of the first few studies in India aimed at identifying the implementation gaps in the P&T policies and systems for MOs from government health department. The paper builds on empirical evidence available on transfer policies and practices in the public health system of India which like other government sectors faces the challenge of corruption [10, 21].

### The health system of the study state

The Department of Health and Family Welfare or the Department of Health is headed by the Minister of Health and Family Welfare while the Principal Secretary of the Health and Family Welfare is the administrative head of the department and responsible for implementing the policies. There are various directorates under the Principal Secretary which are directly involved in implementation of various programmes and activities. The Department of Health and Family Welfare in the state has three directorates (Health, Medical Services and Medical Education) that are mainly responsible for technical as well as administrative support to the health related activities in the state.

The state is divided into six regions with all the 32 districts in the state falling under the six regions. Six Regional Deputy Directors (RDDs), one for each region is incharge for the health related activities for the districts that fall under their region.

At the district level, Chief District Health Officer (CDHO) is the overall incharge of the Community Health Centers (CHCs) and the Primary Health Centers (PHCs) within the district. Similarly all the District Hospitals (DHs) within the district are headed by the Chief District Medical Officer (CDMO) of the district hospital.

DH is Public Hospital that caters to the health needs of the entire district providing mainly tertiary care.

CHC is a 30 bedded hospital that constitutes the secondary level of health care and provide referral as well as specialist health care to the rural population at the block level. It caters to 80,000 - 120,000 population. According to health service norms, each CHC needs to be staffed with specialists as well as regular doctors or MO.

PHC covers a population of 20,000 in hilly, tribal, or difficult areas and 30,000 populations in plain areas with 4–6 indoor/observation beds. It acts as a referral unit for 6 sub-centres and refer out cases to CHC (30 bedded hospital) and higher order public hospitals located at sub-district and district level. Each PHC needs to be staffed with at least one MO.

The MOs have been categorized into two classes: I and II. Both Class I and II are gazetted posts and the state’s Public Service Commission (PSC) called State’s Public Service Commission is responsible for recruitment of all gazetted posts including MOs. All graduate doctors are recruited as Medical Officer (MOs) in Class II to work in Primary Health Centres (PHCs) and/or Community Health Centres (CHCs)) whereas those holding Post Graduate degree in clinical areas are recruited as Specialist as Class I. In addition to specialists, senior level positions at district such as CDHO and CDMO and state level are Class I positions while the MOs working with PHCs and CHCs without Post Graduate specialization are Class II positions.

At the district level, Chief District Health Officer (CDHO) who is a Class I officer is overall in charge of the CHCs and PHCs within the district. Several blocks or the administrative units constitute a district. Blocks are administered by the Block Health Officers (BHOs) who are also usually Class I officers.

Under the compulsory rural service in the state, all the medical graduates from the Government colleges enter the government service under the ‘Bonded’ category and are required to sign a bond at the time of admission to medical college that requires them to compulsorily serve in rural areas for two years.

To address the shortage of MOs in the state, the Dept of Health and Family Welfare in past recruited MOs from such as candidates from private medical colleges or outside the state. Recruitment of such MOs is called Ad hoc appointment. MOs under ad hoc appointment were appointed on a temporary basis and are required to pass the states’ Public Service Commission Exam (PSC) in order to be appointed as permanent employees which would give them regular service.

## Methods

### Study design

The study used qualitative methods first to develop a ‘systems map’ for P&T to identify the extant transfer policies with the help of document review and Key Informant (KI) interviews; then to conduct a systems audit through in-depth interviews with MOs and KIs and construction of job histories to understand how policies are implemented and to identify discrepancies, if any between policies and their actual implementation and the explanations for the discrepancies.

### Study setting

This study was conducted in a Western state in India in 2013. This state was selected for this study as it represents the economically progressive states of India with health indicators much better than the national average, yet the state suffers from shortage of MOs and specialists, especially in rural areas. The vacancy and shortfall in the state is 24 % for MOs at Primary Health Centres (PHCs) while the vacancy and shortfall is particularly high (77 and 93 % respectively) for all specialists working with Community Health Centres (CHCs).

MOs were included as the main respondents of the study working for the government health department placed at rural health centres from three different districts from the state. The study wished to compare transfers to the least and the most desirable posts and therefore selected districts which were categorised, with the help of several MOs and officers working at senior level as “desirable”, “not so desirable” and “not at all desirable”. As several districts were identified in each of the above category, three districts meeting the above criteria were selected from there different regions from the state (out of total six regions) for larger geographical representation.

### Data collection methods and sampling

#### Document review

The current study was part of a larger study that aimed at analysing several other HRM policies other than transfer such as such as recruitment, placement and appraisal. Hence a range of policy documents such as government orders, recruitment rules, appraisal formats etc. were included in the document review. However for the purpose of the current study that focused on transfers, document review of the main policy relating to transfer was done. The transfer policy which is called ‘transfer guideline’ was made available from the government. Content analysis of the same was carried out to understand the extant transfer related rules and policies.

#### Interviews with KI

This group comprised of Informants who occupied key state and district level positions purposively selected for their knowledge of the study topic and to gauge their opinions on the existing policies and how policies are implemented. The study purposively included five KIs to ensure that the views and perspectives of range of stakeholders such as representatives from Health department, administrative department and Medical Association could be represented. Three out of five interviews with KIs were conducted in Hindi (the main language spoken in India) and two interviews in English as two KIs preferred to be interviewed in English) using topic guides. The average time of interviews with KIs was 29 minutes.

#### Interview with MOs

This group consisted of Class I and II MOs who were the main subjects of the study. MOs working mainly with the Primary Health Centres (PHCs) and Community Health Centres (CHCs) were included in the study as these MOs are mainly affected by the policies. During the interviews with MOs, brief job histories were constructed to get deeper insights into transfer processes. The study used purposive sampling at various stages while selecting the study respondents. This purposive selection approach focused on ensuring representation of both male and female doctors; those with Medical graduate degree and/or post-graduate medical degree, both adhoc and bonded doctors, doctors from Block level; as well as regular MOs that were State Public Service Commission (SPSC) confirmed from three different districts representing three different geographical regions from the state were included which would not have been possible through random selection. The total number of interviews with MOs were conducted till the time saturation in information was experienced. Each MO was interviewed only once, making the total number of interviews 19 and all the interviews were conducted with the MOs in Hindi. The average time of interview with MOs was 32.3 minutes.

### Data analysis

Document review analysis: Simple content analysis of the documents was done to understand the existing transfer policies and operational instructions. This included the main policy document called “Revised Guideline for Appointment and transfer of Government employees/officers” [23] or the term ‘transfer guidelines’ used in the paper to refer to the document. The guideline was available in local language of the state) but translated in English for the purpose of analysis.

#### In-depth interviews

All interview recordings were transcribed verbatim and then translated into English. Written consent was sought from study participants and the interviews were audio recorded. Important notes relating to Job histories were also taken during the interviews. Interviews were analyzed using thematic framework approach which is a matrix-based method to arrange and synthesize data [24]. The framework analysis approach was best suited to the scope of current research as the aim of the research was to present themes identified in the data. To analyze the data, study objectives, interview guide and methodology adopted were regularly revisited. The framework approach was used to identify key words, themes and sub-themes and the transcripts of the 24 participants (KIs and MOs) were coded and grouped according to the themes and sub-themes identified. A detailed analysis was performed using NVIVO on the transcribed texts.

#### Job histories

Job histories were constructed from the information available through in-depth interviews with an objective to get deeper insights into the frequencies of transfer and whether transfers complied with the guidelines. Simple descriptive statistics were used to calculate average time for posting (including the first posting) and to compare if such averages complied with transfer rules. The analysis of Job histories also includes the time spent by study respondents during their first posting. This analysis is based for only first posting because the job histories are quite varied where a few respondents had not experienced any transfer or only one transfer while there were other who experienced many transfers. With so much variation in the job histories, it made sense to look at first posting as all the study respondents held at least one posting (the first posting) during their work.

#### Research ethics

Due to the sensitive nature of the study topic that involved interviews with MOs and KIs, the study took great care in maintaining confidentiality of the respondents. The participation in the study was completely voluntary. The ethical approval for the study was sought from institutional ethical review committee at Indian Institute of Public Health Gandhinagar (IIPHG). Relevant permission for the study was also obtained from the Department of Health, from the study state. Further, written consent was obtained from all the MOs and KIs.

## Results

The result section includes the demographic profile of the study respondents. Next, the systems map (extant P&T policies and rules) is explained. Systems audit (to identify practices and implementation of P&T rules and possible discrepancies) is explained next in the section. Several themes were identified based on conceptual framework of the study. Study results have been structured according to the conceptual framework themes: (a) intended HR practices/extant guidelines for transfers; (b) HR practices and implementation of rules. This sub section also contains perceptions of MOs about the implementation of transfers of rules and their reactions or behaviours driven by their perceptions; and (c) knowledge of the MOs about transfer rules which emerged as an important study theme, hence it is another sub head in the results section.

### Demographic details

Of the 19 study respondents, 3 were females while 16 were males. Three respondents were class I officers while 16 were Class II officers. There was almost an equal representation of bonded and adhoc respondents (11 and 8 respectively). As far as place of work is concerned, 7 were from PHCs, 4 each from CHC, DH and BHO (Table 1).

**Table 1:** Distribution of MOs based on demographic and work profile

Class	District 1	District 2	District 3	Total
Class I	1	1	1	3
Class II	4	5	7	16
Gender				
Male	5	5	6	16
Female	0	1	2	3
Place of Work				
PHC	1	3	3	7
CHC	0	2	2	4
DH	3	1	0	4
BHO	1	0	3	4

#### Intended HR practices/extant guidelines for transfers

The transfer guidelines contain several rules, but the present study only focused on two transfer rules as the study respondents were able to share their personal as well as general experiences mainly relating to these two rules, mainly because these two rules were most commonly experienced across all the respondents. The first rule included in the study states that a gazetted officer of Class I and II should not be transferred within 3 years of service and must be compulsorily transferred after 5 years of service at one place. The second rule included in the study suggests that gazetted officers should not use influence of any parliament or legislative assembly member for getting transfer at particular post [25]. More details of these two rules are given in Table 2.

**Table 2:** Various rules under the transfer guidelines

Policy/rule^a^	Explanation of Rule
Rule 1: The 3 and 5 year rule	A gazetted officer of Class I and II should not be transferred within 3 years of service and must be compulsorily transferred after 5 years of service at one place (Government of …….^b^, 2005 Section 1, Page 3)
Rule 2: No use of political influence	If any gazetted officer brings pressure or uses influence of any parliament or legislative assembly member or uses political influence for getting transfer at particular post instead of following proper procedure of getting transfer then the concerned authority should immediately ask explanation from related gazetted officer/employee. (Government of ……,^b^2005 Section 14, Page 13)

^a^The rules presented in the table above have been shortened to authors’ numbering and descriptions

^b^State name has been anonimised

The content analysis of the ‘transfer guideline’ carried out under the study suggested that several transfer related rules have broad conditions under which such rules may not be completely implemented.

One of the KIs suggested that the Health Department has some degree of flexibility to implement the transfer guidelines. However, to what degree such flexibility exists is not clearly stated anywhere in the documents.

“As far as transfer is concerned, there is no separate transfer guideline [for the health sector]. The guidelines issued by Administrative Department is the same for all other officers [from different departments] like accounts, treasury, engineering and for doctors and sometimes these guidelines are not adhered” (KI 1)

“There are transfer rules for all employees- Class I, Class II, Class III as well as Class IV. The government has made policies but as there is shortage of doctors so health department is given some relaxation but such relaxation is not official. Since a doctor works for public so ‘Public interest’ is taken into consideration when such guidelines are applied to health department” (KI 5)

“Health department or any other department can make minor changes in the transfer guideline as per their need……. But if a major change needs to be made in any department then prior approval of administrative department is needed” (KI 5)

#### HR practices and implementation of rules

In this section, we present the job history of the MOs as well as the responses of the KIs and MOs against each of the rule discussed in Table 2 to assess the actual implementation of transfer rules. This section also reflects the perceptions of MOs about the transfer rules and the actual transfer systems and MOs’ reactions driven by perceptions.

##### Rule 1: The 3 and 5 year rule

The job histories of the MOs reveal a total of 73 transfers were experienced by the 19 MOs during their careers with the government. Of these, only 15 (20 %) complied with the Rule 1 (36 months to 60 months rule) while 58 (80 %) of the transfers did not comply with the rule. Of the 58 transfers that did not comply with Rule 1, 44 (76 %) that did not comply with the rule 1 and happened within 3 years (36 months) of posting while the rest 14 happened after 5 years (60 months) of posting at a particular place. The Table 3 gives the details and shows the percentage of respondents who held first posting according to Transfer Rule 1. The job histories indicate that only one fourth of the MOs held their first posting for time period between 3–5 years (as indicated in the 3–5 years rule) while in 75 % of the cases the rule was not followed. Non-adherence of the 3–5 year rule is reflected both for minimum and maximum periods: 42 % of the respondents were transferred within 3 years and 32 % of the respondents experienced transfer after 5 years of service.

**Table 3:** % of Respondents who held first posting for various time periods (*n* = 19)

Post held for Less than 3 years			Post held for 3–5 year	Post held for More than 5 years	
Less than 6 months	6 months to 1 year	1–3 years	3- 5 years	5–7 Years	More than 7 years
2 (11 %)	1 (5 %)	5 (26 %)	5 (26 %)	3 (16 %)	3 (16 %)

**Fig. 2:**
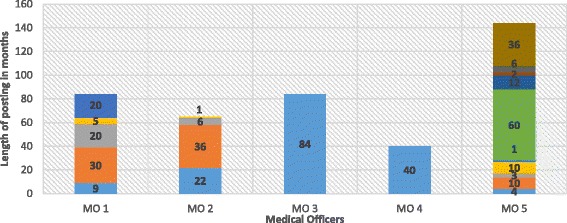
Postings by length and frequency for as sample of MOs. X Axis showing the length of posting held in months. Y Axis showing various postings held by five MOs

Figure 2 illustrates the frequency of transfers with the posting history of sample of the MOs interviewed. It suggests that most of the tenure for postings for MOs does not comply with the 3–5 year (36–60 months) rule. The numeric data presented in Table 3 and Fig. 2 demonstrate that Rule 1 is barely being adhered to.

The responses of KIs and MOs also suggest poor compliance with Rule 1 both in relation to minimum and maximum periods of posting.

“We do have administrative rules that no Class II officer can serve in the same place for more than 3 years and in no event can any officer serve the same place for more than 5 years. Unfortunately that is not implemented. We are having rule that is only on paper and is utilized only when some disciplinary or punitive action needs to be taken against some officers” (KI 4)

“The three year rule it is not strictly implemented” (MO 16)

“MOs can only be transferred after 3 years. However there is no such guarantee. Transfers do happen within 6 months, 9 months and in some cases after 10 years of service” ….There are problems in deployment. It’s not rational and is done haphazardly” (MO 1)

##### Rule 2: No use of political influence

KIs and MOs confirmed the existence of the rule about not using political influence.

“One of the rules is that there should be no political interference and if an MO approaches Political person for his transfer, it is considered as bad behavior and punishable action” (KI 1)

However, from the interviews it appeared that this rule was not normally being followed.

“After health department transfers a MO, he/she has to serve there for 3 years and only then can be transferred again. This is a rule but it doesn’t happen. If you have the right ‘Jugaad’ [Political connection] then you can be transferred within 5 days also. Leave 5 days, your transfer order is renewed the same day” (MO 3)

Most of the KIs and well as the MOs stated that use of political connections is a common way to get favorable transfers. Therefore Rule 2 is some cases in not implemented.

*Reasons and mechanisms for Transfer:* From the interviews with KIs and MOs, the main reasons and mechanisms found under which transfers happened were following:

1. Based on Request of the MO
2. Administrative Reasons or Public Interest
3. Punishment or Complaints against the MO

*Request Based Transfer:* According to transfer guidelines, transfers may be requested by the officials. While such transfers were found to be common, several MOs reported that their requests for such transfers were frequently refused. The study also found that request for transfers sometimes need to be supported with the help of political contact otherwise transfers based on ‘Request’ are not possible.

“I was transferred in March 2008 to Place X as a PHC MO and this transfer was based on request. The transfer happened smoothly on request. ….My most recent transfer also happened on request”. (MO 1)

“There are one or 2 doctors from here who have served for 10–12 years but have not been transferred though they want to be transferred ………..Transfer on request can happen is some cases but in majority cases it doesn’t happen.” (MO 12)

“The most common way of transfer is “approach” [political backing]. At least I do not know any MO who has been transferred on request alone. One has to use some kind of approach else the transfer is not possible” (MO 14)

*Administrative Reasons or Public Interest:* A common reason for transfers is referred to as ‘Admin reasons’. According to one of the KIs, the term ‘Public Interest’ provided a lot of scope for interpretation including political patronage transfers.

“If some transfer happens due to political connections, Government may give it the name of ‘Public Interest’. See the Public Interest term has ambiguity and there are no guidelines that define public interest. For example if a MO is not attending the clinic or not behaving properly then he is transferred in the name of Public interest. But if a politician is requesting for a transfer of MO, this person may be transferred in the name of Public Interest and it cannot be proved. So that’s ambiguity” (KI 1)

The study also found that the exact reasons for transfer labelled ‘Public Interest’ are kept confidential and never disclosed to the concerned MO. The MO would only know that the official reason for the transfer was ‘Public Interest’.

“Public interest includes transfers based on complaints but it’s a confidential thing. Public Interest is something that is not informed to anyone. It’s on file and is not informed to a person. But in transfer order, the government will write transfer based on Public Interest so that MOs can claim dearness and allowance”. (KI 1)

*Punishment based*: A few respondents suggested that MOs may be transferred as a punishment for a variety of reasons. MOs also indicated transfers because of complaints could be reversed if the person had good political connections.

“Yes, transfers happen based on complaints. If there is a complaint against a MO and if the MO does not have any approach [political connection] then such MO could be transferred immediately” (MO 11)

“If there is a complaint against a MO or an enquiry like medical negligence or charging user fee from the patients then such MOs may be transferred” (MO 14)

*Mechanisms for Transfer:* Mechanisms for transfer include a) through mutual transfers (which are legitimate) and two illegitimate mechanisms b) through political connection, and c) involvement of money.

1. *Mutual Transfers:* The study found transfer by mutual agreement between two parties (in this case – MOs) as another form of transfer, although not very common. However how the mutual transfers are treated officially and under which category of Transfer they are categorized is a matter of further exploration.
  “I wanted to go to PHC [x], where a medical officer was working. He did not want to stay there, and was willing to come to my PHC so we went to the Commissioner and it was mutually agreed to interchange our postings” (MO 5)
2. *Political Connections:* Most of the MOs and KIs suggested that use of political connections to get transfers was common. Common synonyms used for ‘political connection’ included: *Jugaad, Jack, Contacts, Approach, Pehchan, Takat*. MOs said that a very common reason for using political connection – for them and colleagues – was to transfer from an undesirable posting or resist transfer from a desirable posting.
  “In my opinion 80 % of the transfers happen due to ‘*Jugaad*’ and 20 due to ‘Request’” (MO 11)
  “I had to put some political pressure otherwise my transfer was not possible- pressure is needed” (MO 13)
  After so much pain, he [another MO the respondent is talking about] used his ‘*Jack*’ [political connection] to get himself transferred to [name of the place]- There are no rules: *‘Jiski Jitni takat and haisiyat’* (Translation: ‘It is a power game’)” (MO 2)
  “Due to political backing transfer takes place within 2 days, or may be someone is posted in morning and he gets a transfer in the evening. I know a doctor who was transferred at 3 different places in the same day.” (MO 7)
  Political influence is sometimes used to actually create vacancies for well-connected MOs. Transfers arranged through political connection are disguised under category of ‘administrative reasons’ or ‘public interest’.
  “Transfers are sometimes done in order to create vacancies for MOs having political contacts” (MO 15)
  “If one has good contacts then he/she can get a good transfer. Transfers happen due to contacts and it’s a common way in our district also. In order to bring someone, others may be removed. And they may be removed under the name of ‘administration need’ saying that 3 years have been completed” (MO 6)
  “There was lot of pressure on us to get transferred ‘on request’. Because if it is not on request then the authorities may require strong reasons to justify the transfer on papers. So we were forced to get transferred ‘On Demand’ to XYZ PHC [name of the PHC changed]” (MO 7)
3. *Use of Money:* The study found that sometimes MOs paid money to get themselves transferred from an undesirable posting or resist transfer from a desirable posting.
  “The main reason for us to be unable to get ourselves transferred is lack of political connections. The only way one can get transferred is either through political connection or through ‘Money Policy’. For example, if you want to go to district X [a desirable district for posting] then the rate is 2 lakh rupees [USD3500]” (MO 8)
  “Sometimes they take money, but mostly the transfers are based on political connections with influence through political leaders and local leaders. For this reason MOs are continuing in one place for many years. But for MO who does not have political connections, money is the next resort” (MO 1)
  “If I wish to be transferred then I can be transferred by tonight. I just need to have 50–60 thousand rupees [USD 1000–1100]…..And people bribe to get transferred, almost 60–80 thousand rupees [USD 1200–1400] for transfers”. (MO 19)
  “Oh yes! Use of Money for transfers is rampant” (MO 16)

#### Knowledge related to transfer related rules

Most of the MOs interviewed demonstrated limited understanding about the transfer rules which was mainly confined to the ‘3 and 5 year’ rule. Further they stated that no specific briefing or guidelines were provided to MOs at any time during their job. Some MOs were unsure which officers had the powers to transfer them. Only four respondents knew that according to the policy that the powers to transfer Class I and II MOs lie with the Health Minister.

“I just know one rule, that once you are transferred you need to serve in that place for at least 3 years. You can only be transferred after 3 years of service. This is the rule made by the government” (MO 11)

“Barring 1 or 2 small rules no MO knows about the transfer rules. One of the rules is that a MO must serve at a place for at least 3 years before being transferred. I don’t know more than this” (MO 17)

## Discussion

Although the study aimed at illuminating the “black box” of transfer systems to find ways of improving staffing, the study only throws a partial light on the issue and has several limitations. While there are many transfer-related rules detailed in the government transfer guidelines, the current study looked at selected transfer related rules experienced by the study respondents. Due to sensitive nature of study topic involving sharing of experiences on illegitimate mechanism used in transfer systems, in a few cases the interviews with MOs reflect the P&T experiences of other MOs rather than their own. Also due to the complexities of the mechanisms of transfers, it was difficult to quantify the number of transfers that happened based on legitimate and illegitimate reasons and mechanisms. In addition due to sensitive and complex nature of the topic, the job histories of the respondents were not validated with the actual transfer orders received by the study respondents. Finally as the study was conducted only in one state based on views and experience on 24 respondents, so generalisations about transfer processes in other states in India cannot be made on the basis of this.

Despite responses from a few KIs suggesting that government understands that transfer needs of health department are different than other government departments, the study found that there are no specific rules for transfer for health department. An earlier study done in India found it surprising that no formal rules exist for transfer of Indian Administrative Services (IAS) officers despite the fact that this cadre is frequently transferred [26]. The results also suggest that the health department has some flexibility to implement the transfer guidelines. However, to what extent such flexibility exists is not clearly laid down in transfer guideline or any other document nor was clearly suggested by any KIs.

The study also found that the transfer rules are not strictly implemented, particularly the rule relating to three and five year transfer. The main explanation of the purpose of the 3–5 year rule is to provide insulation to IAS officers/ civil servants against political pressures from being too frequently transferred [25], but in fact some MOs were using political pressure to get around the rule. Non adherence to such rule has been reported by other studies in India [27] and in the health sector in Pakistan [8]. In the latter case the use of political influence was the main reason why the three year rule was not implemented strictly.

Rule 2 in the civil service in is meant to protect against political influence in transfers, but appears not to be adhered to. In fact it was perhaps one of the most common mechanisms used in transfer. So it can be reasoned that MOs who are not in a position to use political influence for their transfers lose out. A study of doctors in Nepal suggested the use of ‘source force’ – or the use of power and money - was deeply embedded in the social structure and is the institutional modus operandi for HRM in India public administration [14, 15]. In a study done with bureaucrats in India, use of political influence was found to be common [27]. The authors suggest that despite some kind of constitutional insulation available to MOs (in form of the 3 and 5 year rule) against the political pressures, many MOs in fact suffer due to political influence. Hence the overall benefit from this rule-to the health system and the staff who work in it-should be reappraised in future research.

There is no doubt that the transfer guidelines are not being strictly followed; the question is whether this make good sense in terms of human resource management, given the labour market conditions in which the health department is operating. We suggest several reasons for weak implementation of the transfer rules. The transfer rules include very broad and ambiguous conditions under which such rules may not be strictly followed often under ‘Public Interest’ or ‘Administrative reason’. The study findings suggest that many times MOs may be transferred arbitrarily where they either do not know the reasons for their transfer or the reasons known to MOs remained as broad as ‘admin reasons’ or ‘public interest’ as reported by another study done in the same state with IAS officers [28]. When reasons for transfer are used ambiguously, the transparency of the system is lost and then it becomes easier to use political connections in the transfer process. Another possible reason for weak implementation because of the flexibility given to the health sector in the use of generic P&T rules which may sometimes allow certain parallel practices (use of political influence or money) of transfer to take place. While such a flexibility may benefit health systems and certain MOs, it may negatively affect other MOs. Studies elsewhere suggest that in the absence of clear rules and policies, parallel systems emerge [21].

The study results also indicate that the overall transfer system was very unpredictable. Some MOs do not get transferred for fifteen years whereas others are transferred within a few months. For instance we found that 42 % of the MOs were transferred well within 3 years of their first posting and 16 % within the first year of first posting. Similar findings have been reported in India with IAS officers where over half of the IAS officers held their post for less than 1 year with transfers being erratic and frequent movements of officers [29]. Yet another study in India found similar violations in the transfer rules as in the study reported here. The average tenure of IAS officers in a given post is sixteen months and only 56 % of District Officers spend more than one year in their jobs. Such transfers are violation of the recommendations for a three-to five-year tenure in each post put forward by the Ministry of Personnel and the Fifth Pay Commission [25]. Studies done in India on P&T have found that issues of arbitrary transfers may be harassment for government employees [26]. The authors suggest that the unpredictable or arbitrary nature of transfers can demotivate MOs. This was the single most important reason for demoralization and sense of insecurity in a study of IAS officers [26]. The significant link of HR policies and practices on organizational commitment and performance has been discussed in HR management literature [12].

Our study also found that while several MOs fulfilled the required tenure needed under Rule 1 and requested for transfer as per the rule, but it is inferred that there is no one in the health department bound to comply with such request. Studies done on the transfer of IAS officers in India confirm such findings where no one is bound to comply with the legitimate requests of the IAS officers for transfers [28]. Our study also found that the corruption in the P&T process for MOs is not only limited to use of political connections but also found use of money to get favorable transfers. Often such practices allow illicit use of money to have other sources of income as reported by another study done with irrigation department in India which enable rent-seeking for the provision of posts [30]. Also as discussed earlier that while there is a provision where MOs can request for transfer, such requests may not be often granted and the health department need not necessarily entertain such requests. So for the MOs whose legitimate requests for transfer are not granted and for those who cannot break rule number 2 (use of political influence), informal payments remains the last resort.

In order to address the parallel systems that exist in the P&T, our recommendations are in line with La Forgia el al [15] who suggest that the state authorities can enact new and more precise rules governing HR functions as done in the state of Tamil Nadu in India [15]. In addition we also recommend that it is better not to make exceptions to rules – even in the case of the health sector – as this leads to ambiguities. Further, there must be clear definitions about what ‘administrative reasons’ or ‘public interest’ mean to add transparency to the transfer system. However, significant improvements to P&T policies and how they are implemented may not be possible until the external pressure that creates parallel systems is greatly reduced [15].

To address the poor knowledge of MOs about the transfer rules, which is a transparency issue, we recommend improving the information related to transfer rules and their implementation. Similar study in India suggest state-led accountability mechanisms on improving the availability of information on HRM policies and practices that as is underway in some India states such as Tamil Nadu, Karnataka and Odisha [16].

Information about available vacancies, like the one followed in the state of Tamil Nadu in India called ‘Counselling’ (http://www.scribd.com/doc/34652363/Tamil-Nadu-Medical-Service-Counselling-for-transfer-and-promotion-Revised-guidelines-issued#scribd), should be provided to MOs. When fresh recruits are posted to undeserved areas, the length of the posting should be made clear to them and they should be assured that once such a tenure is completed they will be transferred to areas of their choice. Although movement from underserved to preferred locations is not a direct promotion for MOs, but it may be a very powerful way to address the morale of MOs. Such a system exists in Nepal health system where health workers who have served in rural areas may get priority for promotion [31]. The movement from most underserved to preferred locations must be based on availability of vacancies that may be made available to MOs through a systems similar to ‘counselling’ and the requests of the MOs for such transfers. However the health department must ensure that such requests are considered.

## Conclusion

Our study aimed at opening the ‘black box’ of P&T systems in public sector health services in a Western state in India by assessing the implementation gaps between P&T policies and their actual implementation. Results suggest that the transfer guidelines are not implemented robustly, indicating a significant gap between policy and actual implementation. The broad transfer rules and ambiguity in how transfers are treated may explain the discrepancy between policy and implementation leading to systems abuse and corruption.

The study found that overall transfer system for MOs was marked by perceptions of unfairness and irregularities. The P&T system was characterized by de facto institutional mechanisms where the formal transfer rules are undermined by a parallel system in which desirable posts can be attained, retained or sometimes given up (to move to a more desirable place) by use of political connections and in some cases by use of money. Some plausible reasons for poor implementation of P&T policies could be: (1) the flexibility allowed to the health department in implementing transfer rules and absence of any document or common understanding among policy implementers as to what level this flexibility really exists (2) the broad reasons under which transfers are done such as ‘Admin reason’ and ‘Public Interest’.

Effective P&T policies and implementation may have important implications for organizational performance and may help to improve Human Resource (HR) policy and HR expertise. The benefit of the 3–5 year posting rule should be reappraised. Where possible, ambiguity in the rules should be avoided. Greater transparency on implementation of the rules is needed. However, it may not be possible to make any significant improvements to P&T policies and how they are implemented until the external pressure that creates parallel systems is greatly reduced.

## Abbreviations

CHC: Community Health Centre
DH: District Hospital
HRH: Human Resource for Health
HRM: Human Resource Management
KI: Key informants
MO: Medical Officer
P&T: Posting and transfer
PHC: Primary Health Centre
PSC: Public Service Commission
